# BTeam, a Novel BRET-based Biosensor for the Accurate Quantification of ATP Concentration within Living Cells

**DOI:** 10.1038/srep39618

**Published:** 2016-12-21

**Authors:** Tomoki Yoshida, Akira Kakizuka, Hiromi Imamura

**Affiliations:** 1 Department of Functional Biology, Graduate School of Biostudies, Kyoto University, Yoshida-konoe-cho, Sakyo-ku, Kyoto 606-8501, Japan

## Abstract

ATP levels may represent fundamental health conditions of cells. However, precise measurement of intracellular ATP levels in living cells is hindered by the lack of suitable methodologies. Here, we developed a novel ATP biosensor termed “BTeam”. BTeam comprises a yellow fluorescent protein (YFP), the ATP binding domain of the ε subunit of the bacterial ATP synthase, and an ATP-nonconsuming luciferase (NLuc). To attain emission, BTeam simply required NLuc substrate. BTeam showed elevated bioluminescence resonance energy transfer efficiency upon ATP binding, resulted in the emission spectra changes correlating with ATP concentrations. By using values of YFP/NLuc emission ratio to represent ATP levels, BTeam achieved steady signal outputs even though emission intensities were altered. With this biosensor, we succeeded in the accurate quantification of intracellular ATP concentrations of a population of living cells, as demonstrated by detecting the slight distribution in the cytosol (3.7–4.1 mM) and mitochondrial matrix (2.4–2.7 mM) within some cultured cell lines. Furthermore, BTeam allowed continuous tracing of cytosolic ATP levels of the same cells, as well as bioluminescent imaging of cytosolic ATP dynamics within individual cells. This simple and accurate technique should be an effective method for quantitative measurement of intracellular ATP concentrations.

Adenosine 5′-triphosphate (ATP) is the central energy currency of all organisms. It plays critical roles in numerous cellular processes, hence intracellular ATP levels may be closely related to functions, viability, and fate of cells. For instance, assessing cellular ATP contents would be useful for the evaluation of the cytotoxicity and proliferative effects of drugs and various biological compounds^1^,^2^,^3^. Therefore, development of a technique for precise measurement of intracellular ATP levels is imperative.

Conventionally, bioluminescence assay using firefly luciferase has been used for evaluating ATP contents in cell extracts. The luciferase emits light (λ ≈ 550 nm), following hydrolysis of ATP and oxidation of luciferin. This assay typically consists of two steps: extraction of ATP from cells, and reaction of extracted ATP with firefly luciferin-luciferase. However, there are some problems in the conventional assay. First, ATP could be hydrolyzed prior to reaction with luciferase, especially by ATPases released from the cells. Second, bioluminescence output could be affected by variations in several factors, including cell number and luciferin-luciferase concentrations. In addition, the disruptive cell lysis step of this assay makes it impossible to track ATP levels of the same cells over time. Moreover, calculation of intracellular ATP concentration is a complicated task due to the need for precise measurements of both the total cell volume and total amount of ATP.

Efforts have been made to solve the aforementioned problems. For instance, one-step cell homogenization methods could suppress the decomposition of ATP in the extraction process by using modified lysis buffer^3^,^4^. Heterologous expression or introduction of firefly luciferase inside cells enabled the detection of intracellular ATP without lysis of the cells^5^,^6^. More recently, a biosensor based on an ATP-nonconsuming luciferase succeeded in monitoring ATP dynamics inside living cells^7^. The improved methods offer a selection of alternative ATP assays, which provide insights into the dynamics of intracellular ATP. However, these methods still have limitations, including the inability to obtain accurate intracellular ATP levels due to the variable bioluminescence output from luciferases. Furthermore, with the ATP-consuming luciferase-based biosensors, the ATP consumption due to the luciferase-luciferin reaction itself might carry the risk of perturbing the intracellular ATP levels.

Previously, our group developed two types of genetically encoded ratiometric fluorescence biosensors for imaging of ATP levels inside single living cells: Förster resonance energy transfer (FRET)-based ATeam^8^,^9^,^10^, and circularly permuted fluorescent protein-based QUEEN^11^. Different from the luciferase-based ATP assays, the two fluorescence ATP biosensors report ATP level as a ratio value; emission ratio of two fluorescent proteins for ATeam or that of two excitation wavelength for QUEEN. By using the ratio values as output signals, intracellular ATP levels can be quantitatively visualized irrespectively of the expression levels of the biosensors^8^,^9^,^10^,^11^,^12^,^13^. However, those applications are limited due to the necessity of excitation light. For instance, autofluorescence from the background, which is inevitable when using excitation light, potentially causes lower signal-to-noise ratio than bioluminescence techniques, especially in multi-well plate-based assays. In addition, phototoxicity of excitation light to cells or organisms is unavoidable.

In the present study, we developed a novel genetically encoded ratiometric luminescent ATP biosensor, which is based on bioluminescence resonance energy transfer (BRET) and requires no excitation light. With this biosensor we succeeded in measurement of accurate intracellular ATP concentrations in cytosol and the mitochondrial matrix in a population of living mammalian cells. In addition, we demonstrated continuous measurement of intracellular ATP concentrations within the same cells. Of particular note, the biosensor enabled quantitative bioluminescent imaging of intracellular ATP dynamics within individual cells.

## Results

### Development and *In Vitro* Characterization of a BRET-based ATP Biosensor

**Figure 1:**
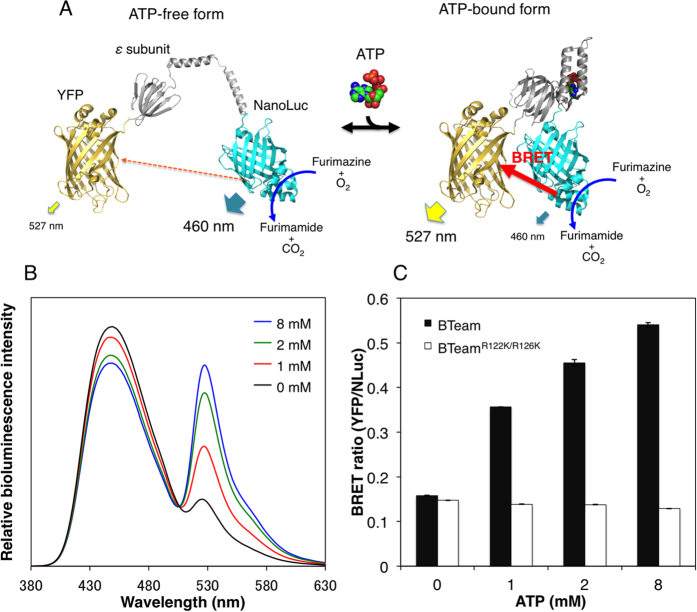
Development of the BRET-based Biosensor BTeam. (**A**) Schematic drawing of BTeam. The ATP-free form of the ε subunit is extended and flexible^16^, which separates YFP and NLuc, resulting in low BRET efficiency (left). On the other hand, binding of ATP to the ε subunit causes a conformational change^16^ that draws YFP and NLuc closer, increasing BRET efficiency (right). It should be noted that the ε subunit reversibly binds and dissociates ATP without its hydrolysis. (**B**) ATP-dependent luminescence spectral changes of purified BTeam. (**C**) ATP-dependent BRET ratio changes of purified BTeam. BRET ratio (YFP/NLuc luminescence ratio, mean ± SD) of purified BTeam (closed bar) and BTeam^R122K/R126K^ (open bar) at different ATP concentrations were calculated from luminometer measurements at 25 °C (*n* = 3, independent repetitions). BTeam^R122K/R126K^ is a variant of BTeam designed not to bind ATP.

We developed a BRET-based ATP biosensor termed BTeam (BRET version of ATeam). BTeam comprises mVenus^14^ (a variant of yellow fluorescent protein, YFP), the ATP binding domain of the ε subunit derived from *Bacillus subtilis* F_o_F_1_-ATP synthase, and NanoLuciferase (NLuc), an ATP-nonconsuming luciferase with characteristics of high pH stability and brightness^15^ (Fig. 1A). BTeam emits lights in the presence of the NLuc substrate, furimazine. As shown in Fig. 1B, BTeam has emission peaks at 455 and 527 nm derived from NLuc and YFP, respectively. BTeam showed elevated BRET efficiency in the presence of ATP, as demonstrated by its emission spectra changes where increase in ATP concentration and YFP intensity is inversely proportional to increase in NLuc intensity. The response of the emission ratios of YFP/NLuc (the BRET ratio) of BTeam could represent ATP concentrations (Fig. 1C). However, the values of the BRET ratio were almost invariant for BTeam^R122K/R126K^, in which the ATP-binding ability was disrupted by replacing Arg-122 and Arg-126 with lysine (Fig. 1C and Supplementary Fig. S1). The results indicate that the BRET ratio is dependent on the conformational changes of the ε subunit^16^ of BTeam upon ATP binding.

**Figure 2:**
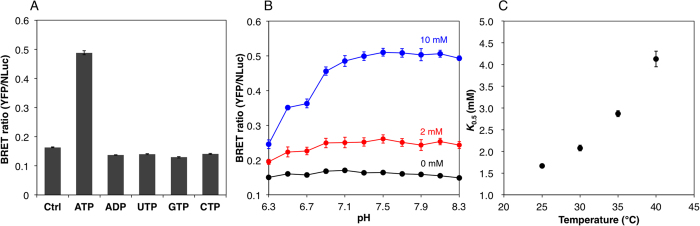
Characterization of purified BTeam. (**A**) Binding selectivity of BTeam to nucleotides. The BRET ratios (mean ± SD) were measured at 37 °C at 0 or 10 mM ATP, ADP, UTP, GTP and CTP, respectively (*n* = 3, independent repetitions). (**B**) pH dependence of BTeam. Effects of pH on BRET ratio (mean ± SD) were investigated at 37 °C at 0–10 mM ATP (*n* = 6 measurements/3 independent repetitions). (**C**) Dependence of ATP affinity on temperature. *K*_0.5_ gives the concentration at which half of the BTeam molecules are bound to ATP. *K*_0.5_ values for ATP were measured at 25, 30, 35, and 40 °C. Mean ± SD values of triplicate measurements are shown.

Next, we examined the chemical properties of purified BTeam (Fig. 2). Among the nucleotides tested (10 mM ATP, ADP, UTP, GTP, or CTP), only ATP increased the BRET ratio of BTeam, while the other nucleotides did not (Fig. 2A). These results are in accordance with previous results regarding ATeam^8^. A slight decrement in the BRET ratio for the other nucleotides was observed, the cause of which remains unknown. However, considering that the decrement was minimal and that those nucleotide concentrations were 3.7–11.3 times lower than that of ATP inside cells^17^, the effects of other nucleotides may be negligible for the ATP measurement with BTeam. BRET ratio of BTeam was almost constant over a range of pH values from pH 7.1 to 8.3, with ATP concentrations ranging from 0–10 mM (Fig. 2B). Previous study has reported that cytosolic and mitochondrial pH is about 7.3 and 8.0, respectively^18^. Consistently, we determined the cytosolic pH of HeLa cell to be 7.34 ± 0.03. Thus, BTeam had appropriate pH stability for evaluating cytosolic and mitochondrial ATP levels. The affinity of BTeam for ATP was weakened with an increase in temperature, as indicated by the values of *K*_0.5_ which demonstrated an exponential increase from 1.7 to 4.1 mM, concurrent with a temperature increase from 25 °C to 40 °C (Fig. 2C). These results suggest that temperature control is crucial for the precise measurement of ATP with BTeam.

### Quantitative Measurement of ATP Levels in Living Mammalian Cells

**Figure 3:**
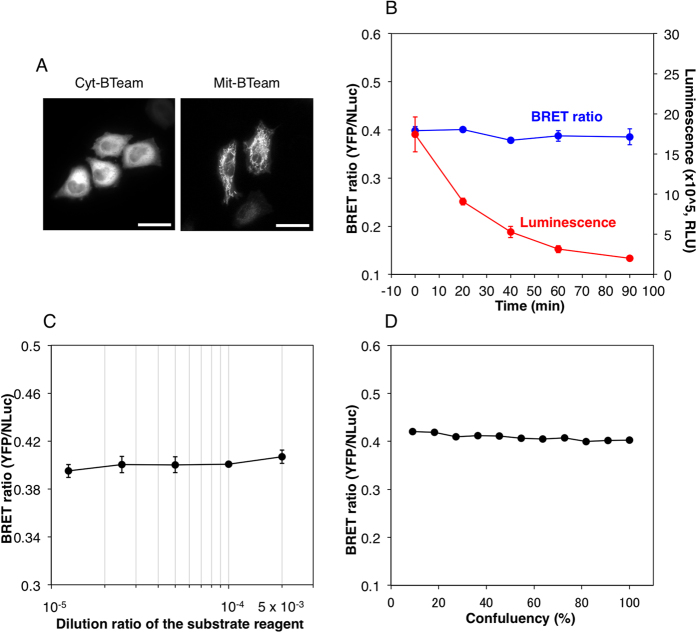
Characterization of BTeam expressed in mammalian cells. (**A**) YFP fluorescence images of HeLa cells expressing cyt-BTeam (left) or mit-BTeam (right). The scale bars represent 30 μm. (**B**) The time course of BRET ratio and luminescent intensity from the HeLa cells stably expressing cyt-BTeam. Mean ± SD values of triplicate measurements are shown. (**C**) Effects of concentration of NLuc substrate reagent in the medium on BRET ratio of HeLa cells stably expressing cyt-BTeam. The BRET ratio of cells at 60–70% confluence was measured in medium containing various concentrations of the substrate reagent. Mean ± SD values of triplicate measurements are shown. (**D**) Effects of cell density on BRET ratio of HeLa cells stably expressing cyt-BTeam. Cell density is given as the relative number of cells in the well (96 well-plate): 1.75 × 10^4^ cells/well represents 100% confluence. A representative data set from multiple independent experiments is shown.

Further characterization of BTeam was carried out at 37 °C using live HeLa cells stably expressing BTeam. When BTeam without any targeting signal sequence (cyt-BTeam) was expressed in the cells, it was localized primarily in the cytosol with a slight fraction in the nucleus, as examined by YFP fluorescence (Fig. 3A). After addition of the NLuc substrate reagent to the medium, luminescence was emitted from the cells. The luminescence intensity decreased as time elapsed. In contrast, the BRET ratio from the cells remained almost constant for 90 min (Fig. 3B). Furthermore, neither the concentration of the substrate reagent in the medium (Fig. 3C) nor the density of the cells (Fig. 3D) affected the values of the BRET ratio from the cells. These characteristics mark a clear improvement over other luciferase-based assays, whose output is sensitive to cell number and luciferin concentrations. These distinct advantages of BTeam as a ratiometric biosensor should translate to more accurate quantification of intracellular ATP levels within living cells. Additionally, the absence of ATP hydrolysis for emission processes of BTeam should allow for more precise evaluation of ATP levels compared to other methods using ATP-consuming luciferases^5^,^6^.

**Figure 4:**
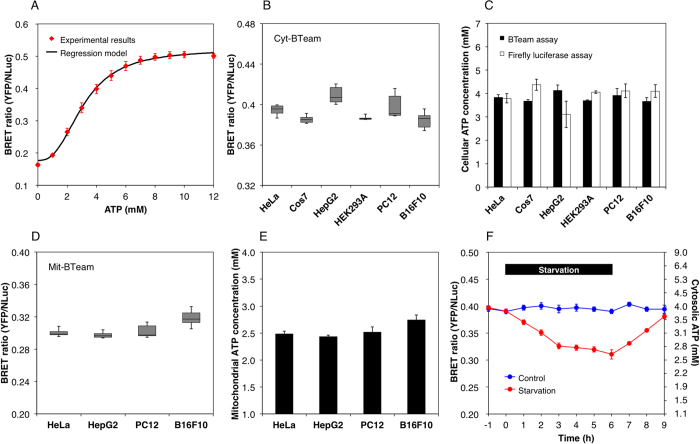
Quantification of intracellular ATP concentrations by using BTeam. (**A**) Standard curve for calculation of ATP concentration. The BRET ratio values (mean ± SD) of purified BTeam at 37 °C were plotted against ATP concentrations, and the regression model was obtained by using Hill equations (*n* = 15 measurements/5 independent repetitions). (**B**) The basal BRET ratios from cells transiently expressing cyt-BTeam. Mean ± SD values of 6 replicate measurements are shown. (**C**) Comparison of intracellular ATP concentrations determined by BTeam (closed bar) and firefly luciferase (open bar). ATP concentrations based on cyt-BTeam were calculated from data in (**B**) using the standard curve (**A**). For ATP determination by firefly luciferase, see Methods section. Mean ± SD values of triplicate measurements are shown. (**D**,**E**) Quantification of mitochondrial ATP concentrations. The basal BRET ratio (**D**) and calculated ATP concentrations (**E**) of cultured cells transiently expressing mit-BTeam. Mean ± SD values of 6 replicate measurements are shown. (**F**) Time-lapse measurements of ATP concentrations within the same cell populations. The BRET ratios of HeLa cells stably expressing cyt-BTeam, which were cultured either in control (blue) or starved (red) conditions, were measured at 1 h intervals. The cells were cultured in phenol red-free DMEM containing 5% FBS. At 0 h, starvation was initiated by exchanging the medium with EBSS containing 5% FBS and 1.0 g/L glucose. After 6 h starvation, the medium was re-exchanged to phenol red-free DMEM containing 5% FBS. A representative data set is shown. Error bars represent SD (*n* = 10 wells in 96 plate). Results from 2 independent experiments worked consistent.

Next, we demonstrated the measurement of basal intracellular ATP levels in several cell lines with BTeam (Fig. 4). Cells transiently expressing BTeam were incubated in a 96-well plate, and the basal intracellular ATP levels at 60~70% confluence were measured. A regression model using Hill’s equations was obtained, based on the BRET ratios of purified BTeam versus ATP concentrations at 37 °C as a standard curve for the calculation of ATP concentrations (Fig. 4A). The BRET ratio values were in the range from 0.385 to 0.409 (Fig. 4B). The calculated intracellular ATP concentrations were 3.8 ± 0.1 mM in HeLa, 3.7 ± 0.1 mM in Cos7, 4.1 ± 0.2 mM in HepG2, 3.7 ± 0.04 mM in HEK293A, 3.9 ± 0.3 mM in PC12, 3.7 ± 0.2 mM in B16F10 cell lines (Fig. 4C). ATP concentrations calculated from BTeam measurements were nearly equal to those estimated by a conventional firefly luciferase assay (*see* Materials and Methods for detail). The calculated ATP concentrations are also consistent with the previously reported values (2.5 ± 1.2 and 3.1 ± 2.1 mM for normal and tumor cells, respectively)^17^. Considering these results, BTeam exhibits promising potential as an accurate and effective tool for quantitative measurement of intracellular ATP concentrations. ATP levels in the mitochondrial matrix were also measured with mit-BTeam, a variant BTeam targeted to the mitochondrial matrix (Fig. 3A and Supplementary Fig. S1). The values of the BRET ratio from four transiently transfected cell lines were in a narrow range from 0.297 to 0.318 (Fig. 4D). The calculated mitochondrial ATP concentrations were 2.5 ± 0.1 mM in HeLa, 2.4 ± 0.03 mM in HepG2, 2.5 ± 0.1 mM in PC12, and 2.7 ± 0.1 mM in B16F10 cells (Fig. 4E). Mitochondrial ATP levels were approximately 1.5 mM lower than cytosolic levels, a consistent trend reported in previous studies^8^,^19^,^20^,^21^,^22^. A potential explanation for the disparity between cytosolic and mitochondrial ATP levels is a rapid exchange of ATP and ADP between the cellular compartments via adenine nucleotide translocator. Additionally, ADP is a primary building block for ATP synthesis, and evidence of higher mitochondrial ADP levels has been previously reported^17^. Our results further corroborate the difference between cytosolic and mitochondrial ATP levels.

**Figure 5:**
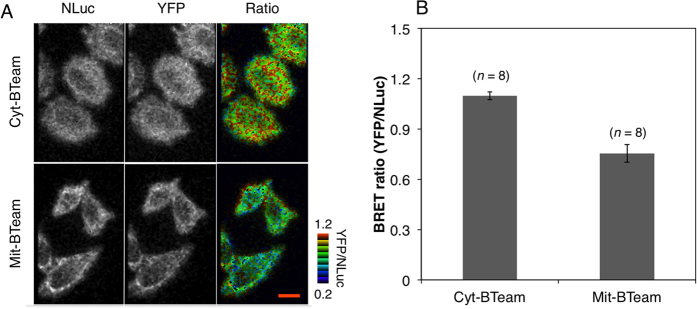
Visualization of ATP levels in cytosol and mitochondria of single HeLa cells. (**A**) Luminescence images of NLuc (left) and YFP (middle), and BRET ratio (right, pseudocolored) of HeLa cells stably expressing cyt-BTeam (upper) or transiently expressing mit-BTeam (bottom). The red scale bar represents 8 μm. (**B**) Comparison of BRET ratio values between cytosol and mitochondria. The values were calculated from each single HeLa cell. The numbers of cells used for calculating the ratio are indicated. Error bars represent standard deviation.

We used BTeam to trace changes in cytosolic ATP levels of the same cells. It was clearly evident that the BRET ratio of HeLa cells stably expressing BTeam gradually decreased in the starved condition, and rapidly recovered after replenishment of nutrients (Fig. 5E). The result indicates that BTeam is adequate for continuous measurements of accurate intracellular ATP of the same cells.

### Ratiometric Luminescent Imaging of Intracellular ATP Levels of Single Living Cells

**Figure 6:**
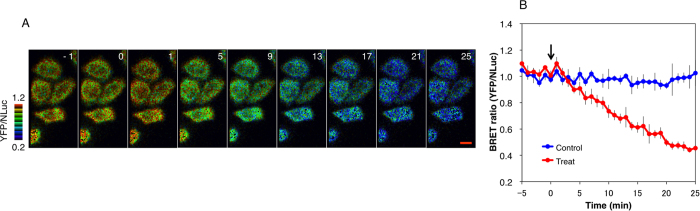
Time-lapse BRET imaging of cytosolic ATP levels in living HeLa cells. (**A**) Sequential pseudocolor images the BRET ratio of HeLa cells stably expressing cyt-BTeam. Intracellular ATP depletion in the cells was monitored after addition of 3 μg/mL oligomycin A and 20 mM 2-deoxyglucose at time = 0 (min). The numbers in the figure represent the time (min) after addition of the inhibitors. The scale bar represents 8 μm. (**B**) Time-course of the BRET ratio of HeLa cells stably expressing cyt-BTeam. The blue and red lines represent the ratios obtained from control cells and cells treated with inhibitors, respectively. Error bars represent standard deviation (*n* = 8 individual cells). The arrow indicates the time of addition of the inhibitors to the medium.

Finally, ATP levels in individual living cells were imaged with BTeam using a microscope. NLuc and YFP emissions from a BTeam-expressing cell were separated with a beam splitter and were simultaneously captured with an EMCCD camera. Even at single-cell level, the BRET ratio of the cytosol was significantly higher than that of mitochondria (Fig. 5C,D), consistent with the data obtained from a population of cells using a luminometer (Fig. 4). It should be noted that, due to the filter settings, the different basal values in the BRET ratio of the cells were observed between imaging assays and luminometer assays (*see* Material and Methods for details). We then imaged cytosolic ATP levels of single living cells over time. When 2-deoxyglucose and oligomycin A, inhibitors of glycolysis and oxidative phosphorylation, respectively, were added to the medium, a rapid decrease in the BRET ratios of the cells was observed (Fig. 6A,B and Supplementary video 1). The result indicates that BTeam is not only adequate for quantitative measurement of intracellular ATP of a population of cells, but also is capable of exploring ATP dynamics at the single-cell level.

## Discussion

In the present study, we developed a novel BRET-based ATP biosensor, termed BTeam. BTeam presents ATP concentrations as a value of the BRET ratio (emission ratio of YFP/Nluc). Based on this property, BTeam can steadily measure ATP even though emission intensities are altered, unlike other bioluminescent ATP assays. With this biosensor, we demonstrated accurate measurements of intracellular ATP concentrations of a population of living cells, as well as longitudinal monitoring of cytosolic ATP levels of the same cells. The intracellular ATP levels measured with BTeam were nearly equal to those estimated from cell extracts using firefly luciferase assay, which was consistent with previous report^17^. Our results further corroborate the importance of intracellular ATP levels in the health conditions of cells, since assessing ATP contents in cell extracts has been used for potential explanations of the cytotoxicity and proliferative effects of drugs^1^,^2^,^3^,^4^. Due to its simple and accurate properties for measurements of cytosolic and/or mitochondrial ATP of multiple cell populations, BTeam would be useful for high-throughput drug screenings that result in changes in ATP levels.

Fluorescence biosensors have recently been applied to imaging of ATP levels, or the ATP/ADP ratio, in living cells^8^,^9^,^10^,^11^,^23^. In general, temporal and spatial resolutions are better in imaging with fluorescence biosensors than with luminescent biosensors because fluorescent biosensors can emit stronger signals. However, BTeam may also have some advantages in live cell ATP imaging over fluorescence-based biosensors. For instance, BTeam may be potentially useful for investigating ATP dynamics in plants, because unlike fluorescence biosensors it does not require excitation light, which would perturb photosynthetic activity and induce strong autofluorescence from chlorophyll. In addition, BTeam may be used in conjunction with optogenetics techniques that utilize light-sensitive actuators such as channelrhodopsin^24^, which could be skewed by excitation light for traditional fluorescent biosensors.

In conclusion, BTeam is as an effective tool for accurate measurement of intracellular ATP concentrations, and it should be useful in a wide variety of applications.

## Methods

### Chemicals and Cells

Cells were routinely cultured at 37 °C in 5% CO_2_ in the following growth medium: Dulbecco’s modified Eagle’s medium (DMEM) with 10% fetal bovine serum (FBS) for HeLa, Cos7, HEK293A, and HepG2; DMEM with 10% FBS and 5% horse serum for PC12; and Minimum Essential Media (MEM) with 10% FBS for B16F10 cells. Cells expressing BTeam were generated by transfection with plasmids carrying BTeam cDNA, using Lipofectamin^®^2000 reagent (Invitrogen). The transfected cells were assayed between 1 and 3 days after transfection. To establish stable cell lines expressing BTeam, cells were cultured in growth medium containing 0.75 mg/mL G418 for over 2 weeks after transfection. Subsequently, cells stably expressing BTeam were isolated. All chemical reagents and other solvents used were analytical grade. Nucleotides, including ATP, were pre-mixed with equimolar magnesium chloride before use; the term ATP in the present experimental results represents Mg-ATP.

### Gene Construction

A modified cDNAs for mVenus, with an in-frame deletion eliminating 11 amino acids from the C-termius, and NLuc (Promega) with a deletion of 3 amino acids from the N-terminus (with permission from Promega), were amplified by PCR, respectively. The PCR products were ligated into the XhoI-ClaI and EcoRI-HindIII sites in pRSET-AT1.03^8^, respectively, to generate pPRSET-BTeam for expression of BTeam in *Escherichia coli.* The cDNA of BTeam was excised from pRSET-BTeam with XhoI and HindIII (Thermo Scientific). The restriction fragment was then ligated into the XhoI-HindIII sites of pcDNA3.1 (−) (Invitrogen) for mammalian expression. The constructs used in the present study are illustrated in Supplementary Fig. S1.

### Purification of BTeam

The BTeam protein was purified with a MagneHis^TM^ Protein Purification kit (Promega) with minor modifications. *E. coli* strain Rosetta (DE3) carrying the BTeam plasmid was cultured in 2 × YT medium at 25 °C for 40–60 h. Cells were collected by centrifugation at 3,200 × *g* for 5 min and re-suspended in 10 mM PBS (pH 7.4) containing protease inhibitors (cOmplete Mini EDTA-free tablet, Roche). The suspension was disrupted by sonication, and centrifuged at 20,000 × *g* at 4 °C for 10 min. The supernatant was applied to MagneHis^TM^ Ni-Particles pre-equilibrated with PBS. After two washes with the manufacturer’s wash buffer, the purified BTeam protein was eluted with 200 mM imidazole. The purified proteins were stored at 4 °C, and used within 2 days after purification.

### Characterization of Purified BTeam

Luminescence spectra of the purified BTeam were investigated at 25 °C in 50 mM Mops-KOH buffer (pH 7.3), containing 50 mM KCl, 0.5 mM MgCl_2_, and 0.05% Triton X-100 (Buffer A), using a FP-6500 spectrofluorometer (Jasco). Luminescence spectra from 370 to 720 nm at 2000 nm/min of scanning speed were immediately scanned after addition of 2 uL Nano-Glo^®^ Luciferase Assay Substrate (Promega) to a 400 uL reaction solution of BTeam and 0–8 mM ATP.

Emissions of YFP and NLuc from purified BTeam were measured for 0.5 sec using an ARVO-X3 (PerkinElmer) equipped with the following emission filters (Semrock): FF01–515/LP for YFP emission; and FF01–450/70 for NLuc emission. After addition of the substrate reagent to a reaction mixture composed of purified BTeam and 0–12 mM ATP in Buffer A, the BTeam YFP/NLuc emission ratio was measured at 25–40 °C. For measurements at higher temperatures (>25 °C), the sample plate was incubated in the pre-heated plate reader for 15 minutes, followed by addition of the substrate and a further 15 minutes incubation prior to measurement. The nucleotide binding selectivity of BTeam was investigated at 37 °C with 10 mM ATP, ADP, UTP, GTP and CTP, respectively, in Buffer A. The pH dependency of BTeam was investigated at 37 °C at 0–10 mM ATP in buffers containing 50 mM Mops-KOH (pH 6.3–7.5) or Hepes-KOH (pH 7.7–8.3), 50 mM KCl, 0.5 mM MgCl_2_, and 0.05% Triton X-100. The temperature dependence of the ATP affinity (*K*_0.5_) of BTeam was investigated at 25–40 °C with a range of 0–12 mM ATP in Buffer A. The values of *K*_0.5_ were calculated by using Hill equations; R = (R_max_ − R_min_) × [S]^n^/([S]^n^ + *K*_0.5_^n^) + R_min_, where n is the Hill coefficient, R_max_ and R_min_ are the maximum and minimum BRET ratios, respectively.

### Luminometer assays

Cells expressing BTeam were seeded into a 96-well plate, and incubated for 24–36 h at 37 °C in 5% CO_2_ in growth medium. After washing with DMEM containing 5% FBS, cells were incubated for 30 min in phenol red-free DMEM containing 5% FBS, 20 μM HEPES (pH 7.5), and 30 μM NLuc inhibitor to avoid disturbance from the BTeam released from dead cells. After addition of NLuc substrate reagent to the medium, microplates were incubated at 37 °C for 20 min to stabilize the temperature in a CO_2_ incubator. Then, luminescence emissions from the cells were measured at 37 °C for 0.5 sec using an ARVO-X3 equipped with the same emission filter set for the assay of the purified BTeam as described above. Figure 4 (B,D) illustrates the basal BRET ratio of cells transiently expressing cyt-BTeam or mit-BTeam measured 30–36 h after passage around 70% confluency. To calculate ATP concentrations from the BRET ratios of the cells, a regression model was obtained by using Hill equations for standard curve. In this study, the following coefficient values were used for the standard curve; 0.521 for R_max_, 0.178 for R_min_, 3.11 for *K*_0.5_, and 2.58 for n, respectively, which were calculated from the plots of the BRET ratio values of purified BTeam against ATP concentrations at 37 °C.

Total cellular ATP determination with firefly luciferase was carried out using a firefly luciferase kit (Toyo B-net) with slight modifications. Cells were trypsinized, and suspended in growth medium. Aliquots of the suspension were collected by centrifugation at 6,000 × *g* for 3 min at 4 °C. Pellets were disrupted with Glo Lysis Buffer (Promega) for 5 min followed by centrifugation at 20,000 × *g* for 5 min at 4 °C. ATP concentration in the supernatant was quantified with the reagents provided in the kit. The cellular ATP levels were calculated using the following equation: Cellular ATP level = [ATP]/(CN × CV), where [ATP] and CN represent the ATP amount and cell numbers in the aliquot, respectively, and CV represents cell volume estimated from the diameters of the trypsinized cells. Cell numbers and diameters were measured using a CDA-500 particle analyzer (Sysmex).

### Microscopy assay

The transfected HeLa cells were plated on a collagen coated glass bottom dish, and subjected to imaging. Prior to imaging, the medium was changed to phenol red-free DMEM containing 5% FBS and 30 μM NLuc inhibitor (Promega), and the cells were incubated for 20 min at 37 °C in 5% CO_2_. BRET imaging was immediately carried out after addition of the substrate reagent to the medium. Imaging was performed on a Nikon Eclipse Ti-E inverted microscope with a perfect focus system (Nikon Instruments) using a CFI Plan Apo VC 60x oil-immersion objective lens, 1.40 numerical aperture (Nikon Instruments). Dual-luminescence emission ratio imaging was achieved by using a W-View Gemini optical splitting device (Hamamatsu Photonics) equipped with FF01–450/70 (Semrock) for the NLuc emission filter and FF01–542/27 for the YFP emission filter. Luminescence emission from BTeam was obtained with a 5 sec exposure time using an EMCCD camera (Andor Technology). During imaging, the cells were maintained at 37 °C in 5% CO_2_ on a microscope by using a top-stage incubator (Tokai Hit). Analysis of images was performed using MetaMorph (Molecular Devices). The YFP/NLuc emission ratio was calculated by using the integrated intensity of YFP emission and NLuc emission within the area of the whole cell (cytosol) or the area surrounding the mitochondria inside individual cells, respectively. The ATP levels were illustrated as pseudo color images generated by the emission ratio of YFP/NLuc calculated from YFP and NLuc luminescence images.

### Cytosolic pH measurement

The cytosolic pH of HeLa cells was measured with BCECF (2′-7′-bis(carboxyethyl)-5(6)-carboxyfluorescein) as the following protocol. The cells were treated with 10 μM BCECF acetoxymethyl ester (Molecular probes) in Hank’s balanced salt solution (HBSS) for 15 min at 37 °C, followed by washing with HBSS twice. Fluorescence intensity of BCECF of the cells (*n* = 6) was measured at 37 °C in DMEM using a Nikon TE2000 (Nikon Instruments) equipped a CFI Plan Apo VC 60x oil-immersion objective lens, 1.40 numerical aperture (Nikon Instruments). The following filters (Semrock) were used for the measurement; excitation filters (FF01-445/20 and FF01-482/18), an emission filter (FF01-520/35), and a dichroic mirror (FF495-Di03). Images were captured with an ORCA-AG cooled CCD camera (Hamamatsu photonics). Calibration curve was obtained by imaging BCECF-loaded cells in pH-controlled buffers containing 150 mM KCl, 20 mM NaCl, 0.5 mM MgCl_2_, 0.5 mM CaCl_2_, 10 mM MES, 10 mM HEPES, 10 μM monensin and 10 μM nigericin.

## Additional Information

**How to cite this article**: Yoshida, T. *et al*. BTeam, a Novel BRET-based Biosensor for the Accurate Quantification of ATP Concentration within Living Cells. *Sci. Rep.* **6**, 39618; doi: 10.1038/srep39618 (2016).

**Publisher's note:** Springer Nature remains neutral with regard to jurisdictional claims in published maps and institutional affiliations.

## Supplementary Material

Supplementary Information

Supplementary Movie 1
