# Supplementary material for: BTeam, a Novel BRET-based Biosensor for the Accurate Quantification of ATP Concentration within Living Cells
Source: Sci Rep. 2016 Dec 21;6:39618. doi: 10.1038/srep39618 (PMC5175186; doi:10.1038/srep39618)
Supplement: Supplementary Information [file srep39618-s1.pdf]

Supplementary Information

BTeam, a Novel BRET-based Biosensor for the Accurate Quantification of ATP Concentration within Living Cells

Tomoki Yoshida, Akira Kakizuka and Hiromi Imamura

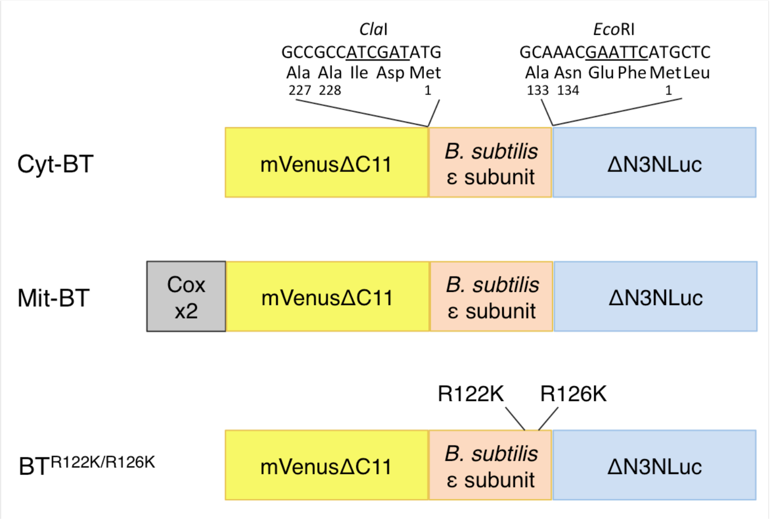

Fig. S1. Gene constructs used in this study.

BTeam (cyt-BT) is composed of mVenus with a deletion of 11 amino acids from the C-terminus, *Bacillus subtilis* ε subunit with V9T/V42K/F67N/L78T mutations, and NLuc with a deletion of 3 amino acids from the N-terminus. Two-amino acid spacers were used to connect mVenus and the ε subunit, and the ε subunit and NLuc, respectively. Mit-BT has a pair of mitochondrial targeting signals from cytochrome *c* oxidase subunit VIII at the N terminus. $BT^{R122K/R126K}$ has R122K/R126K double mutations in the ε subunit domain of BTeam.

Supplemental video 1. BRET imaging of cytosolic ATP levels of living HeLa cells.

Intracellular ATP depletion in HeLa cells stably expressing cyt-BTeam was monitored after addition of 3 μg/mL oligomycin A and 20 mM 2-deoxyglucose at time = 0 (min). Intracellular ATP levels are displayed as the BRET ratio in pseudo-color.
